# Patient-centered outcomes and interaction effects in a factorial trial of pneumoperitoneum pressure and neuromuscular blockade depth

**DOI:** 10.1016/j.bjane.2026.844758

**Published:** 2026-04-23

**Authors:** Eylem Yasar, Melike Korkmaz Toker

**Affiliations:** a Mugla Research and Training Hospital, Department of Anesthesiology and Reanimation, Mugla, Turkiye; b Mugla Sıtkı Koçman University, Faculty of Medicine, Department of Anesthesiology and Reanimation, Mugla, Türkiye

Dear Editor,

We read with great interest the randomized, double-blind factorial study by Meletti et al., recently published in Brazilian Journal of Anesthesiology, examining the combined influence of pneumoperitoneum pressure and neuromuscular blockade depth on postoperative recovery after laparoscopic cholecystectomy.^1^ By deliberately placing postoperative quality of recovery at the center of their analysis through the use of the validated Quality of Recovery (QoR)-40 questionnaire, the authors move beyond a purely technical intraoperative focus toward outcomes that are directly meaningful to patients. This perspective is particularly relevant in contemporary perioperative practice. At the same time, several methodological and interpretative aspects deserve closer examination, as they may influence how the reported neutral findings should be understood.

In trials using a factorial design, interpretation relies heavily on whether potential interactions between interventions are formally evaluated. In the present study, pneumoperitoneum pressure and neuromuscular blockade depth are intentionally combined within a 2 × 2 framework; however, the results are primarily presented as group-wise comparisons, without explicit assessment of a pressure-neuromuscular blockade interaction. This aspect of the study design is methodologically relevant. Several observations suggest that the effect of neuromuscular blockade depth may vary according to pneumoperitoneum pressure rather than acting uniformly across conditions. Notably, deep neuromuscular blockade under standard pneumoperitoneum pressure was associated with improved surgical field conditions but also with longer post-anesthesia care unit stay, higher resting pain scores at 24 hours, and increased use of rescue antiemetics. In the absence of formal interaction testing, it is difficult to determine whether these findings reflect independent effects, context-dependent interactions, or random variation. Specifically, the published report does not present an interaction term, a p-value for interaction, or model-based factorial estimates assessing whether the effect of neuromuscular blockade depth differs across pneumoperitoneum pressure levels. Outcomes are instead reported as group-wise comparisons across the four study arms. When factorial designs are used to evaluate combined intraoperative strategies, failure to explore interaction effects may weaken conclusions regarding the absence of benefit. At a minimum, reporting a pressure × neuromuscular blockade interaction term with its corresponding p-interaction, or providing model-based estimates stratified by pressure level, would allow readers to distinguish between independent and context-dependent effects.

The contrast between surgeon-rated and patient-reported outcomes is particularly striking. The improvement in surgical field quality observed with deep neuromuscular blockade is consistent with prior randomized trials and meta-analyses demonstrating superior laparoscopic working conditions under deep blockade compared with moderate blockade.^2^ Current European guidelines similarly endorse the use of deep neuromuscular blockade to optimize surgical exposure when visualization is limited.^3^ However, the present findings also illustrate that technical optimization does not automatically translate into improved patient experience. Despite better intraoperative conditions, patients receiving deep neuromuscular blockade under standard pneumoperitoneum pressure experienced less favorable postoperative outcomes. In procedures such as laparoscopic cholecystectomy, where hospital stay is short and recovery expectations are high, even modest increases in pain, nausea, or recovery time may influence patient satisfaction and the perceived value of care. These observations therefore merit consideration rather than being readily dismissed as clinically insignificant. These secondary outcome differences should be interpreted as hypothesis-generating, particularly in the context of multiple comparisons and the absence of adjustment for multiplicity.

Interpretation of the neutral QoR-40 results also deserves a more nuanced approach. The study was powered to detect a 10-point difference in QoR-40 scores, whereas the most widely accepted evidence-based estimate for the minimum clinically important difference of this instrument remains approximately 6.3-points, derived using anchor- and distribution-based methods in perioperative populations.^4^ Although this approach was established prior to 2020, it continues to be widely used as a benchmark for interpreting clinical relevance in QoR-40 – based studies and has not undergone major revision in subsequent validation work. It should be acknowledged that estimates of the minimum clinically important difference may vary according to surgical setting, patient population, and timing of assessment. More recent research on postoperative recovery has increasingly focused on abbreviated instruments such as the QoR-15, largely because of their psychometric strength and feasibility in contemporary cohorts.^5^ This shift in the literature highlights a key methodological issue: powering a study to detect an effect size substantially larger than the accepted clinically meaningful threshold increases the likelihood of concluding equivalence when smaller, yet patient-relevant, differences in global recovery may exist. From this perspective, the absence of statistically significant differences in QoR-40 scores should be viewed as evidence against a large early effect, rather than as definitive proof that pneumoperitoneum pressure or neuromuscular blockade depth has no meaningful influence on postoperative recovery.

In the published trial, eight of 132 randomized patients (approximately 6%) were excluded after randomization, with analyses conducted per protocol. While modest in magnitude, such post-randomization loss may still influence the interpretation of patient-reported outcomes, particularly when intention-to-treat analyses are not applied. Beyond these design considerations, the role of pharmacological reversal also warrants attention. In this trial, the deep Neuromuscular Block (NMB) group received 4 mg·kg^-1^ sugammadex, whereas the moderate NMB group was reversed with neostigmine and atropine. Given the faster and more predictable recovery profile of sugammadex, as well as its distinct side-effect profile compared with acetylcholinesterase inhibitors, differences in post-anesthesia care unit stay and postoperative symptoms such as nausea and resting pain may be confounded by the choice of reversal agent rather than reflecting the isolated effect of neuromuscular blockade depth.

Overall, the trial by Meletti et al. offers important insight into the complex relationship between pneumoperitoneum pressure, neuromuscular blockade depth, and early postoperative recovery. Nevertheless, careful interpretation of its neutral results requires attention to factorial design principles, clinically meaningful effect thresholds, and the potential trade-offs between technical optimization and patient-centered outcomes. Moving forward, intraoperative management strategies must be evaluated not just by technical success, but by their ability to harmonize surgical optimization with the quality of the patient's early functional recovery.

## Institutional Review Board (IRB) approval

Not applicable.

This correspondence does not report original patient data and is based on the critical appraisal of a previously published study.

## Study registration

Not applicable.

No new clinical study was conducted as part of this correspondence.

## Authors’ contributions

Conceptualization: Eylem Yasar, Melike Korkmaz Toker.

Critical analysis and interpretation: Eylem Yasar, Melike Korkmaz Toker.

Drafting of the correspondence: Eylem Yasar.

Critical revision for intellectual content: Melike Korkmaz Toker.

Final approval of the manuscript: All authors.

## Funding

None.

## Data availability statement

No new data were created or analyzed in this study. Data sharing is not applicable to this article.

## Declaration of competing interest

The authors declare no conflicts of interest.
